# Introduction, Spread and Impact of the SARS-CoV-2 Omicron Variants BA.1 and BA.2 in Cyprus

**DOI:** 10.3390/microorganisms10091688

**Published:** 2022-08-23

**Authors:** Jan Richter, Dana Koptides, Christina Tryfonos, Denise Alexandrou, Christina Christodoulou

**Affiliations:** 1 Molecular Virology Department, Cyprus Institute of Neurology and Genetics, Nicosia 2371, Cyprus; 2 Medical and Public Health Services, Ministry of Health, Nicosia 1148, Cyprus

**Keywords:** SARS-CoV-2, COVID-19, Omicron, whole-genome sequencing, epidemiology, Cyprus

## Abstract

The aim of this study was to investigate and obtain insights into the appearance, spread and impact of the Omicron variants and their sub-lineages in Cyprus by analyzing 611 high-coverage full-genome sequences for the period from November 2021 until April 2022. All viruses sequenced were identified to belong to either Delta (B.1.617.2) or Omicron (lineage BA.1 and BA.2, respectively), with a variety of different sub-lineages. A detailed analysis of the mutational profile is presented and discussed. The Omicron variant BA.1 was shortly followed by BA.2; despite emerging against a background of high vaccination (81% of adult population) and pre-existing natural immunity, they gave rise to the largest waves of infection, with daily numbers rising dramatically, highlighting their increased ability for immune evasion. Within a period of only five months, the percentage of the Cypriot population with a confirmed infection increased from ~15% of the total population to >57%. Despite unprecedented case numbers, a significant reduction in hospital burden and mortality was observed. Our findings highlight the role of the importation of new variants through travel and demonstrate the importance of genomic surveillance in determining viral genetic diversity and the timely identification of new variants for guiding public health intervention measures.

## 1. Introduction

Two and a half years after the beginning of the COVID19 pandemic caused by the newly emerged Severe Acute Respiratory Syndrome Coronavirus 2 (SARS-CoV-2), more than 550 million confirmed cases and 6,350,000 deaths have been reported worldwide (https://covid19.who.int/, accessed on 27 June 2022). SARS-CoV-2 first appeared in Wuhan, China in December 2019, becoming the seventh coronavirus currently known to infect humans [1]. The four seasonal endemic human coronaviruses (CoVs) cause mild to moderate disease and belong to the Alphacoronavirus genus (HCoV-NL63, HCoV-229E) and the Betacoronavirus genus (HCoV-OC43, HCoV-HKU1), respectively [2]. In addition, the genus Betacoronavirus includes MERS-CoV and SARS-CoV, which have emerged in the last 20 years and cause severe disease and even possibly fatal acute respiratory distress syndrome (ARDS) [3]. Coronaviruses are enveloped, positive-sense, single-stranded RNA viruses of ~30 Kb genome size, placing them among the largest RNA viruses known [4]. Their mutation rate is estimated to be lower than that of other RNA viruses as they have a unique proofreading mechanism mediated by the NSP14 protein, which exhibits 3′ to 5′ exoribonuclease activity [5]. In addition, NSP14 may have a critical role in RNA recombination events during viral replication that can generate genetic variants [6].

WHO, in collaboration with partners, expert networks, national authorities, institutions and researchers, has been monitoring and assessing the evolution of SARS-CoV-2. Emerging variants are named in a non-stigmatizing manner using Greek alphabet letters and are classified as variants of concern (VOCs), variants of interest (VOIs) or variants under monitoring (VUMs) based on the risk posed to global public health. Circulating SARS-CoV-2 variants are regularly assessed in terms of transmissibility, disease severity, risk of reinfection, impacts on diagnostics and vaccine performance and are reclassified accordingly. To date, five variants have been classified as VOCs: Alpha, Beta, Gamma, Delta and the most recent Omicron. The Delta variant (B.1.617.2) was first detected in India in October 2020 and dominated globally until late 2021 [7], when the Omicron variant (B.1.1.529) appeared and rapidly became the prevalent variant worldwide.

The Omicron variant was first detected in specimens from Botswana and South Africa on 11 and 14 November 2021, respectively [8]. It was reported to WHO on 24 November and designated as a VOC within only two days. When the first sequences of the Omicron full genome became publicly available, they revealed that it has the highest number of alterations across its genome compared to the original Wuhan Hu-1 strain of all known variants [9]. Notably, more than 30 amino acid substitutions, three deletions and one insertion are located in the coding region of the spike protein, half of them within the receptor-binding domain (RBD), the primary target of neutralizing antibodies (NAbs) [10]. Considering coding and noncoding genomic regions, Omicron carries at least 60 mutations compared with the Wuhan strain. Many of these mutations have been observed in previously described VOCs, whereas some are exclusively found in Omicron [11]. Several hypotheses have been put forward for the emergence of the Omicron variant, including evolution in an immunocompromised individual, long-term circulation in an area with low surveillance or through reverse anthroponosis from non-human species reservoirs [12,13].

The Omicron variant consists of several lineages, with the main lineages being BA.1 and BA.2. At first, BA.1 was detected circulating in most countries, but BA.2 quickly replaced it and was classified as a VOC independently of BA.1. A number of studies and surveillance data, including in Cyprus, indicate a reduction in disease severity and hospitalization frequency with BA.1 and BA2 variant infections compared to Delta, despite the increased transmissibility of the former [14,15,16,17]. Additionally, reduced susceptibility of the BA.1 and BA2 variants to neutralizing antibodies induced by previous SARS-CoV-2 infection or vaccination is reported [18,19].

In a previous study, we investigated the molecular epidemiology of SARS-CoV-2 in Cyprus from the beginning of the pandemic until the emergence of the Alpha VOC in January 2021 [20,21]. Distinct lineages of SARS-CoV-2 that drove the first three major waves of infections reflective of the epidemiological pattern were identified. The aim of this study is to investigate and obtain insights into the appearance, spread and impact of the Omicron variants and their sub-lineages in Cyprus in the period from November 2021 until April 2022.

## 2. Materials and Methods

### 2.1. Sample Collection and Next-Generation Sequencing

The Department of Molecular Virology of the Cyprus Institute of Neurology and Genetics was assigned as the reference laboratory for SARS-CoV-2 by the Cyprus Ministry of Health of the Republic of Cyprus. During the study period of November 2021 until April 2022, approximately 110,000 samples from public health services were received and analyzed, from which 14,190 were positive for SARS-CoV-2. Following an agreement of the Government with ECDC, 96 samples were sent on a bi-weekly basis to Eurofins Genomics Europe Sequencing GmbH for full-genome sequencing. For sample selection, a random, unbiased approach was taken without pre-screening for variants of interest to avoid sampling bias. The viral RNA had been previously detected using the Thermo Fisher TaqPath™ COVID-19 CE-IVD RT-PCR kit and all samples selected showed a cycle threshold value (Ct) lower than 30. The study has been approved by the Cyprus National Bioethics Committee (EEBK 21.1.01.03). According to the approval, the Bioethics Committee waived the requirement for informed consent as samples were completely anonymized.

### 2.2. Lineage Assignment and Mutation Analysis

Following quality control filtering, 611 high-coverage full-genome sequences were selected for the period November 2021 until April 2022. For variant identification and mutation calling, the dynamic nomenclature tool Phylogenetic Assignment of Named Global Outbreak Lineages (PANGOLIN) version 4.1.1 (https://pangolin.cog-uk.io, accessed on 1 June 2022) [22] as well as the COVID-19 genome annotator (http://giorgilab.unibo.it/coronannotator, accessed on 3 June 2022) [23] were employed. Bubble charts were created in R v4.2.0 with ggplot2 packages [24]. All sequences obtained were deposited at the GISAID EpiCov database, with accession numbers EPI_ISL_13773508-EPI_ISL_13774143 (www.gisaid.org, accessed on 12 July 2022).

### 2.3. Phylogenetic Analysis

Phylogenetic trees were constructed using IQ-Tree v2.2.0 [25], employing the maximum likelihood method and ultrafast bootstrapping with 1000 replicates. JModelTest v2.1.10 [26] was used for testing and determining the best-fitting nucleotide substitution model applying Bayesian information criterion scores, which yielded GTR + I + G as the best-fitting substitution model. Trees were visualized using the interactive Tree of Life Tool (iTOL) v6.5.7 [27].

## 3. Results and Discussion

**Figure 1:**
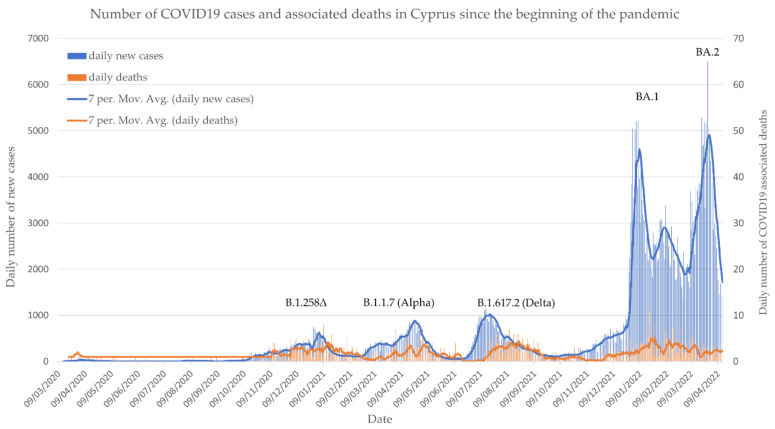
Number of daily positive SARS-CoV-2 cases as well as the number of recorded COVID-19-associated deaths in Cyprus since the detection of the first case in March 2020 until April 2022 (https://www.data.gov.cy/, accessed on 30 May 2022).

From the detection of the first case in March 2020 until April 2022, the Republic of Cyprus recorded 474,105 SARS-CoV-2 confirmed cases (53% of the population) along with 1015 associated casualties (113/100,000) (https://www.data.gov.cy/, accessed on 30 May 2022). As shown in Figure 1, the SARS-CoV-2 pandemic had progressed in Cyprus through several sequential waves, with each major wave initiated usually by the emergence of a new phenotypically distinct variant of concern. At the beginning of the period under study here, i.e., November 2021, the B.1.617.2 lineage was dominating, and with the onset of the colder season, case numbers had started to rise slowly. By the end of December, however, an unprecedented sharp increase in cases per day was observed, exceeding 5000 per million people, which was caused by the Omicron variant BA.1 lineage. This wave peaked in the first week of January 2022, after which cases declined rapidly. At the beginning of February, a minor increase was observed that subsided by the beginning of March; however, numbers started to rise sharply again, culminating in the largest peak so far in the first week of April, after which numbers started to drop steadily.

A similar pattern was observed in many countries around the world, where Delta and its sub-lineages had been the predominant variants during the last few months of 2021. Within a short period between the end of December 2021 and the first few weeks of January 2022, the percentage of Delta rapidly dropped, being replaced by the Omicron variant (see Supplementary Materials Figure S2) [28,29,30]. The emergence of the Omicron variant and its rapid spread was reportedly accompanied by a massive increase in case numbers, with the United Kingdom, for example, reporting record numbers of COVID-19 infections, with daily reported case numbers approaching 200,000 [31].

### 3.1. Lineage Analysis

For the period from November 2021 until April 2022, 611 high-coverage full-genome sequences that passed stringent quality control criteria were included in the sequence analysis. The results of the lineage assignment by PANGOLIN are summarized in Table 1. All viruses sequenced belonged to one of two variants of concern, namely Delta (B.1.617.2) or Omicron (lineage BA.1 and BA.2, respectively). Within the Delta lineage, 17 different sub-lineages were distinguished, with AY.43, AY.122 and AY.4 being the most frequently encountered. The AY.43 lineage was identified predominantly in Europe (http://cov-lineages.org, accessed on 4 July 2022), with Greece, Germany and France reporting the highest frequencies [32].

AY.122, characterized by the combination of mutations nsp2:K81N and Orf7a:P45L, was reported to be the predominant Delta variant in Russia [33] and also the Seychelles during the respective waves [34], even though no clear fitness advantage could be associated with the specific defining mutations [33]. In Cyprus, AY.122’s frequency was at the highest at the beginning of November and dropped thereinafter against the background of the increasing diversity of Delta sub-lineages.

The BA.1 and BA.2 lineages identified consisted of 14 and 6 sub-lineages (Table 1), respectively, with BA.1.1, BA.1, BA.1.17.2 and BA.2, BA.2.9 being the most common Omicron sub-lineages encountered.

**Figure 2:**
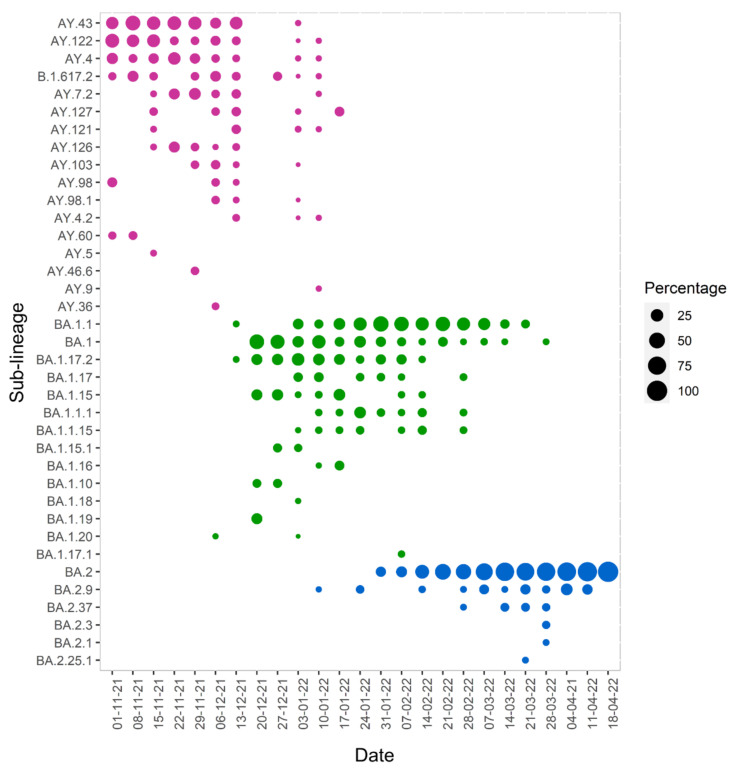
Bubble chart illustrating the weekly frequency of SARS-CoV-2 sub-lineages over time. The size of the circle is proportional to the percentage of each sub-lineage detected each week.

In the bubble chart in Figure 2, the weekly frequency and diversity of the different SARS-CoV-2 sub-lineages over time are illustrated in more detail. From the figure, it can be seen that the most frequent sub-lineages both of Delta and Omicron were detected throughout the study period with varying diversity over time.

**Figure 3:**
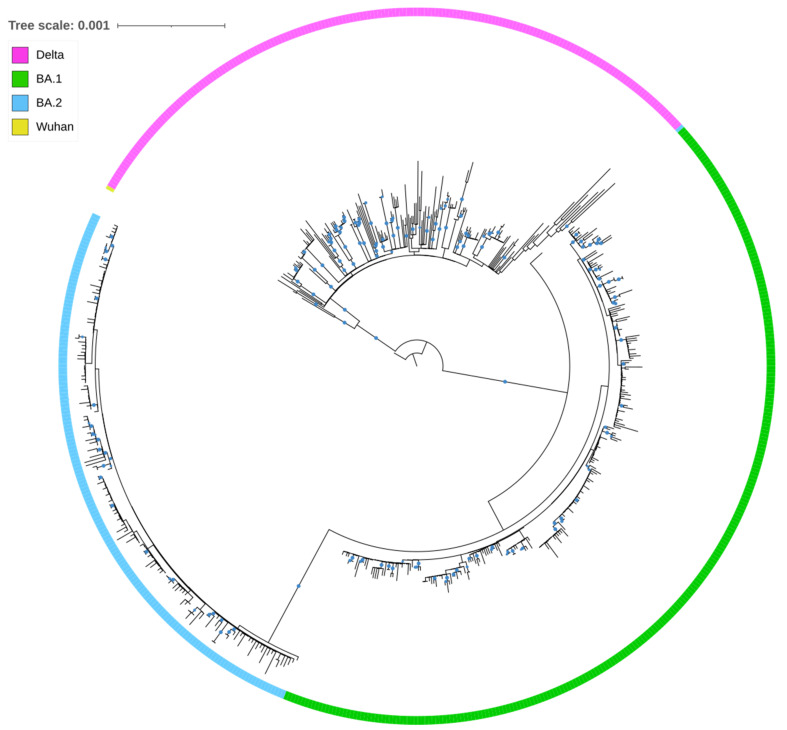
Phylogenetic tree of Cypriot full-genome sequences (n = 611) analyzed between November 2021 and April 2022 aligned against the reference genome hCoV-19/Wuhan/Hu-1/2019 (NC_045512.2). Bootstrap values >80% indicate the percentages of replicate trees, in which the associated taxa clustered together in the ultrafast bootstrap test (1000 replicates) are indicated by blue circles.

Figure 3 shows the result of the phylogenetic analysis, which is in support of the PANGOLIN lineage assignment and reveals a picture of many independent, distinct importations that were followed by local transmission.

**Figure 4:**
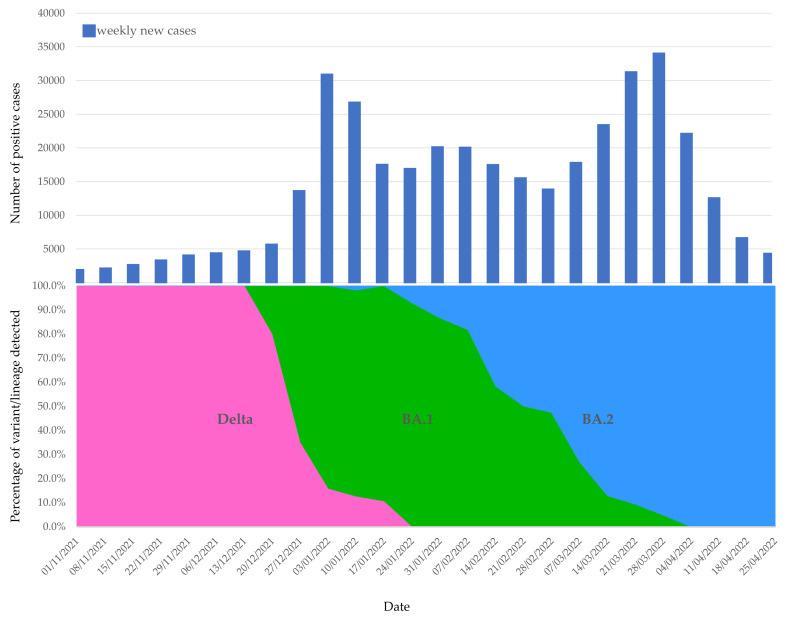
Time course of the frequency of the SARS-CoV-2 VOC identified in Cyprus in relation to the number of daily positive cases recorded between November 2021 and April 2022.

In Figure 4, the weekly prevalence of each VOC of concern is depicted in conjunction with the number of positive cases detected. From the graph, it is evident that the sharp increase in positive cases at the end of December 2021 was caused by the introduction and subsequent spread of the Omicron BA.1 variant. This variant was detected for the first time in Cyprus on December 10, and within less than one month, it became the dominant variant, by the first week of January 2022. Shortly after, the BA.2 variant was identified for the first time, which gained steady ground and replaced the BA.1 variant as the dominant one by the beginning of March 2022, giving rise to the largest wave so far, with record daily numbers of new cases.

### 3.2. Mutation Analysis

Overall, 838 and 715 SNPs were identified within the Delta and Omicron sequences, respectively, when compared to the Wuhan-Hu-1 reference genome. For more details, Supplementary Tables S1 and S2 list all SNPs identified in the Delta and Omicron variants that were observed with a frequency >50% in at least one sub-lineage. Table 2 summarizes and compares the spike protein mutations identified in the Delta and Omicron lineages that were observed with at least 10% frequency. Only the T478K and D614G mutations are shared between all three lineages. The D614G mutation was acquired already very early in the pandemic, as it enhances SARS-CoV-2 replication in the upper respiratory tract through increased virion infectivity [35], while the effect of T478K is less well understood. In addition, all three lineages possess a mutation at position 681. In the case of Delta (P681R), this mutation was shown to lead to improved furin cleavage efficiency of full-length spike to S1 and S2, and thereby more efficient virus fusion entry into respiratory epithelial cells [36], even though this mutation was found only in 53% of the Delta sequences, with minor differences among sub-lineages (Supplementary Materials Table S1).

In contrast, the P681H mutation in conjunction with N679K and H655Y in the Omicron variants BA.1 and BA.2 appears to result in suboptimal S1/S2 cleavage and an inability to use the transmembrane serine protease 2 (TMPRSS2), which is required for the activation of the spike protein to facilitate membrane fusion, and impaired syncytium formation compared to the Delta variant [37]. Omicron appears to favor the endosomal entry route, which does not require spike cleavage and favors the infection of cells of the upper respiratory tract [38], resulting in a reduction in severity compared to Delta. In addition, the L452R mutation in the receptor-binding motif (RBM), which is one of the defining Delta mutations but is absent in BA.1 and BA.2, has been associated not only with resistance to some monoclonal antibodies [39] but also with T-cell immunity escape [40]. In a recent study, where the L452R mutation was introduced in a Omicron BA.1 variant, it enhanced the ability of Omicron to infect the lung tissues of humanized mice [41]. The L452R mutation indeed was acquired later by the BA.2.12.1 sub-lineage of BA.2 and is also present in the latest BA.4/BA.5 VOCs. While preliminary in vitro and in vivo data suggest that BA.4/5 and BA.2.12.1 replicate more efficiently in human lung cells compared to BA.2 [42], and experiments in hamsters suggest that BA.4 and BA.5 may cause more severe disease, reports from South Africa suggest that the risk of severe hospitalization/death was similar during the BA.4/BA.5 and BA.1 waves [43]. Nonetheless, these latest variants (BA.2.12.1, BA4 and BA.5) showed further augmented neutralization resistance against vaccine-induced antibodies [44].

In comparison with the Delta VOC, it was shown that the increased number of mutations located in the Omicron N-terminal domain, such as T95I and V213G, critically modify exposed epitopes, hindering their recognition by non-structural domain-targeting neutralizing antibodies, thereby permitting reinfection and reducing the efficacy of previous vaccinations [45,46]. Moreover, mutations S371L, N440K, G446S and Q493R in the S protein confer greater antibody resistance to Omicron compared to previously circulating VOCs [47].

It is noteworthy that a significant number of mutations found in Omicron (T95I, G142D, K417N, T478K, N501Y, D614G, H655Y and P681H) overlap with the previous VOCs Alpha, Beta, Gamma and Delta [48]. These overlapping mutations have been previously associated with increased transmissibility, more efficient viral binding, as well as immune evasion.

Despite the increased transmissibility of Omicron, a number of studies and surveillance data have shown a marked reduction in disease severity and hospitalization frequency compared to Delta, a trend that could also be discerned in Cyprus [49,50,51]. Supplementary Figure S1 shows the significant reduction in the case fatality rate that coincided with the appearance of the BA.1 lineage at the end of 2021 and continues to fall until the end of April 2022, when BA.2 became the dominant variant.

## 4. Conclusions

The Omicron SARS-CoV-2 variant of concern (Pango lineage B.1.1.529) was first detected in South Africa and Botswana in November 2021 [8]. Following its rapid spread, intensive research efforts to characterize this VOC quickly demonstrated signs of increased transmissibility and high potential for immune evasion [52,53], but simultaneously lower virulence compared with the previously dominating Delta variant [15,16]. In addition, several characteristics of the virus appear to have changed, among which are the potential to infect human cells through endocytosis, as well as a pronounced tropism for the upper respiratory tract [37,54,55]. After South Africa, the Omicron variant spread quickly and caused epidemic waves in many countries [56]. By the end of March 2022, the Omicron variant had been detected in more than 180 countries and had become dominant on a global scale, accounting for 99.7% of submitted sequences (https://covid.cdc.gov/covid-datatracker/#variant-proportions, accessed on 4 July 2022).

Here, in this study, we have described the changing pattern of circulating SARS-CoV-2 lineages in Cyprus between November 2021 and April 2022. The Omicron lineage BA.1 emerged at the beginning of December 2021 and rapidly replaced Delta against a background of high vaccination (81% of adult population) and pre-existing natural immunity, giving rise to the largest wave of infections, with daily numbers rapidly rising, confirming its increased ability for immune evasion and reflecting the selection pressure exerted by previous vaccination and natural infection. It was shortly followed by the BA.2 lineage in a pattern that was observed in many countries around the world. Within a period of only five months, the percentage of the population with a confirmed infection increased from ~15% of the total population to >57% (www.data.gov.cy, accessed on 30 May 2022). Despite soaring case numbers, a significant reduction in hospital burden and mortality was observed.

The continuous surveillance of SARS-CoV-2 by whole-genome sequencing remains critical for the timely detection of emerging variants, to identify major transmission modes, as well as to guide public health intervention.

## Figures and Tables

**Table 1:** Frequency of sub-lineages detected in the study period.

WHO Label	Lineage	Sub-Lineage	Number	% of VOC
Delta	B.1.617.2	AY.43	45	24.3%
		AY.122	29	15.7%
		AY.4	21	11.4%
		B.1.617.2	18	9.7%
		AY.7.2	14	7.6%
		AY.127	13	7.0%
		AY.121	9	4.9%
		AY.126	7	3.8%
		AY.103	7	3.8%
		AY.98	6	3.2%
		AY.98.1	5	2.7%
		AY.4.2	4	2.2%
		AY.60	2	1.1%
		AY.36	2	1.1%
		AY.5	1	0.5%
		AY.46.6	1	0.5%
		AY.9	1	0.5%
		Total	185	
Omicron	BA.1	BA.1.1	81	30.5%
		BA.1	59	22.2%
		BA.1.17.2	49	18.4%
		BA.1.17	20	7.5%
		BA.1.15	16	6.0%
		BA.1.1.1	12	4.5%
		BA.1.1.15	11	4.1%
		BA.1.15.1	6	2.3%
		BA.1.16	3	1.1%
		BA.1.10	2	0.8%
		BA.1.18	2	0.8%
		BA.1.19	2	0.8%
		BA.1.20	2	0.8%
		BA.1.17.1	1	0.4%
		Total	266	
Omicron	BA.2	BA.2	133	83.1%
		BA.2.9	15	9.4%
		BA.2.37	8	5.0%
		BA.2.3	2	1.3%
		BA.2.1	1	0.6%
		BA.2.25.1	1	0.6%
		Total	160	

**Table 2:** Comparison of the frequency of spike protein mutations identified in the three VOCs in Cyprus. Only mutations with a frequency >10% in either variant are shown (NTD: non-structural domain, RBD: receptor-binding domain, RBM: receptor-binding motif, FP: fusion peptide, HR1: heptad repeat 1 region. A color gradient corresponding to the frequencies of SNPs between 0% and 100% was applied to highlight the most frequently occurring SNPs.

Position			SNP	Delta	BA.1	BA.2
21595	**S1**	SP	V11V	2.7%	12.4%	0.0%
21618		NTD	T19I	0.0%	0.0%	98.1%
21618			T19R	100.0%	0.0%	0.0%
21762			A67V	0.0%	100.0%	6.8%
21846			T95I	39.5%	92.1%	6.8%
21987			G142D	99.5%	0.0%	100.0%
22200			V213G	0.0%	0.0%	100.0%
22578		RBD	G339D	0.0%	92.1%	100.0%
22599			R346K	0.0%	38.3%	0.0%
22673			S371L	0.0%	99.6%	0.0%
22674			S371F	0.0%	0.4%	100.0%
22679			S373P	0.0%	100.0%	100.0%
22686			S375F	0.0%	100.0%	100.0%
22688			T376A	0.0%	0.4%	100.0%
22775			D405N	0.0%	0.4%	100.0%
22786			R408S	0.0%	0.4%	100.0%
22792			I410I	0.5%	0.0%	11.2%
22813			K417N	0.0%	100.0%	100.0%
22882		RBM	N440K	0.0%	100.0%	100.0%
22898			G446S	0.0%	100.0%	0.0%
22917			L452R	100.0%	0.0%	0.0%
22992			S477N	0.0%	98.5%	100.0%
22995			T478K	100.0%	98.5%	100.0%
23013			E484A	0.0%	98.5%	100.0%
23040			Q493R	0.0%	98.5%	100.0%
23048			G496S	0.0%	98.5%	0.0%
23055			Q498R	0.0%	98.5%	100.0%
23063			N501Y	0.0%	98.5%	100.0%
23075			Y505H	0.0%	98.5%	100.0%
23202			T547K	0.0%	95.1%	0.0%
23403			D614G	100.0%	99.2%	100.0%
23525			H655Y	0.5%	90.6%	95.7%
23599			N679K	0.0%	86.5%	94.4%
23604			P681H	0.0%	86.1%	93.8%
23604			P681R	53.5%	0.0%	0.0%
23664	**S2**		A701V	0.0%	17.7%	0.0%
23854			N764K	0.0%	100.0%	100.0%
23948		FP	D796Y	0.0%	100.0%	100.0%
24130			N856K	0.0%	97.0%	0.6%
24410		HR1	D950N	100.0%	0.0%	0.0%
24424			Q954H	0.0%	100.0%	100.0%
24469			N969K	0.0%	100.0%	100.0%
24503			L981F	0.0%	100.0%	0.0%
25000			D1146D	0.0%	100.0%	100.0%

## Data Availability

All sequences obtained were deposited at the GISAID EpiCov database with accession numbers EPI_ISL_13773508-EPI_ISL_13774143 (www.gisaid.org).

## Supplementary Materials

The following supporting information can be downloaded at: https://www.mdpi.com/article/10.3390/microorganisms10091688/s1, Table S1: Frequency of SNPs observed in Delta sub-lineages in Cyprus; Table S2: Frequency of SNPs observed in Omicron sub-lineages in Cyprus; Figure S1: Case fatality rate in Cyprus from November 2021 until April 2022. Figure S2: Percentage of SARS-CoV-2 sequences that are Delta and Omicron variants, respectively, in selected countries from November 2021 until April 2022.
