# Supplementary material for: Introduction, Spread and Impact of the SARS-CoV-2 Omicron Variants BA.1 and BA.2 in Cyprus
Source: Microorganisms. 2022 Aug 23;10(9):1688. doi: 10.3390/microorganisms10091688 (PMC9503937; doi:10.3390/microorganisms10091688)
Supplement: Supplementary file 1 [file microorganisms-10-01688-s001.zip › Supplementary Figure S1.pdf]

Figure S1. Case-fatality rate (CFR) in Cyprus from November 2021 until April 2022.

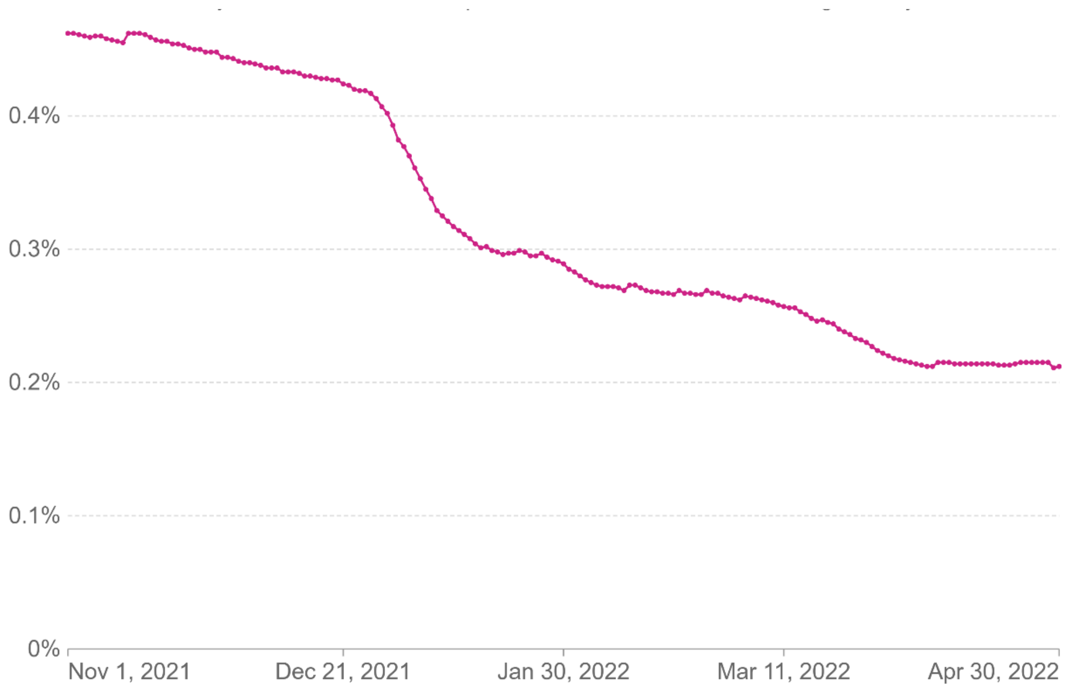

Source: https://ourworldindata.org/explorers/coronavirus-data-explorer.
