# Supplementary material for: Introduction, Spread and Impact of the SARS-CoV-2 Omicron Variants BA.1 and BA.2 in Cyprus
Source: Microorganisms. 2022 Aug 23;10(9):1688. doi: 10.3390/microorganisms10091688 (PMC9503937; doi:10.3390/microorganisms10091688)
Supplement: Supplementary file 1 [file microorganisms-10-01688-s001.zip › Supplementary Table S1.pdf]

Table S1. Mutations identified in the Delta sub-lineages in Cyprus between November 2021 and April 2022. Only SNPs with frequency >50% were included. Mutations with frequency >75% are marked in green, SNPs with frequency between 50% and 75% are marked in yellow.

Position	Number	Variant	protein	AY.43	AY.122	AY.4	B.1.617.2	AY.7.2	AY.127	AY.121	AY.126	AY.103	AY.98	AY.98.1	AY.4.2
210	185	210	5'UTR	100%	100%	100%	100%	100%	100%	100%	100%	100%	100%	100%	100%
241	185	241	5'UTR	100%	100%	100%	100%	100%	100%	100%	100%	100%	100%	100%	100%
526	10	E87D	NSP1	0%	0%	0%	0%	0%	0%	0%	0%	0%	83%	100%	0%
664	6	G133G	NSP1	0%	0%	0%	0%	0%	0%	0%	0%	86%	0%	0%	0%
835	14	F10F	NSP2	0%	0%	0%	0%	100%	0%	0%	0%	0%	0%	0%	0%
854	9	P17S	NSP2	0%	0%	0%	50%	0%	0%	0%	0%	0%	0%	0%	0%
1048	29	K81N	NSP2	0%	100%	0%	0%	0%	0%	0%	0%	0%	0%	0%	0%
1210	5	M135I	NSP2	0%	0%	0%	0%	0%	0%	0%	0%	0%	0%	100%	0%
1274	9	V157I	NSP2	0%	0%	0%	50%	0%	0%	0%	0%	0%	0%	0%	0%
1306	6	E167D	NSP2	0%	0%	0%	0%	0%	0%	0%	0%	0%	100%	0%	0%
1594	13	S263S	NSP2	0%	0%	5%	0%	0%	92%	0%	0%	0%	0%	0%	0%
2144	14	V447L	NSP2	0%	0%	0%	0%	100%	0%	0%	0%	0%	0%	0%	0%
2447	5	G548C	NSP2	0%	0%	0%	0%	0%	0%	0%	0%	0%	0%	100%	0%
2518	9	V571V	NSP2	0%	0%	0%	0%	0%	0%	0%	0%	0%	67%	100%	0%
2638	5	L611L	NSP2	0%	0%	0%	0%	0%	0%	0%	0%	0%	0%	100%	0%
2944	14	L75L	NSP3	0%	0%	0%	0%	100%	0%	0%	0%	0%	0%	0%	0%
3037	185	F106F	NSP3	100%	100%	100%	100%	100%	100%	100%	100%	100%	100%	100%	100%
3821	6	L368F	NSP3	0%	0%	0%	0%	0%	0%	0%	0%	86%	0%	0%	0%
4181	182	A488S	NSP3	100%	100%	100%	100%	100%	100%	100%	100%	100%	100%	100%	100%
5437	5	E906D	NSP3	0%	0%	0%	0%	0%	0%	0%	0%	0%	0%	100%	0%
5570	6	M951V	NSP3	0%	0%	0%	0%	0%	0%	0%	0%	86%	0%	0%	0%
6710	6	V1331F	NSP3	0%	0%	0%	0%	0%	0%	0%	0%	0%	100%	0%	0%
6712	6	V1331V	NSP3	0%	0%	0%	0%	0%	0%	0%	0%	86%	0%	0%	0%
7124	171	P1469S	NSP3	93%	93%	95%	89%	100%	92%	89%	100%	100%	83%	100%	100%
7768	5	I1683I	NSP3	0%	0%	0%	0%	0%	0%	0%	0%	0%	0%	100%	0%
7851	25	A1711V	NSP3	0%	0%	100%	0%	0%	0%	0%	0%	0%	0%	0%	100%
7923	9	S1735F	NSP3	0%	0%	0%	50%	0%	0%	0%	0%	0%	0%	0%	0%
8179	14	R1820R	NSP3	0%	7%	0%	0%	0%	92%	0%	0%	0%	0%	0%	0%
8476	9	N1919N	NSP3	0%	0%	0%	50%	0%	0%	0%	0%	0%	0%	0%	0%
8727	13	A58V	NSP4	0%	0%	0%	0%	93%	0%	0%	0%	0%	0%	0%	0%
8733	9	T60I	NSP4	0%	0%	0%	50%	0%	0%	0%	0%	0%	0%	0%	0%
8986	181	D144D	NSP4	100%	100%	100%	100%	100%	100%	89%	100%	100%	100%	100%	100%
9053	72	V167L	NSP4	31%	34%	29%	56%	43%	62%	56%	14%	43%	50%	60%	50%
9190	10	V212V	NSP4	0%	0%	0%	0%	0%	77%	0%	0%	0%	0%	0%	0%
9805	6	L417L	NSP4	0%	0%	0%	0%	0%	0%	0%	0%	0%	100%	0%	0%
9889	12	L445L	NSP4	0%	0%	0%	0%	0%	92%	0%	0%	0%	0%	0%	0%
10029	179	T492I	NSP4	98%	93%	100%	100%	100%	100%	100%	100%	100%	100%	100%	100%
10291	6	G79G	NSP5	0%	0%	0%	0%	0%	0%	0%	0%	0%	100%	0%	0%
10507	12	N151N	NSP5	0%	0%	0%	0%	0%	92%	0%	0%	0%	0%	0%	0%
11201	182	T77A	NSP6	100%	100%	100%	100%	100%	100%	100%	100%	100%	100%	100%	100%
11332	182	V120V	NSP6	100%	100%	100%	100%	100%	100%	100%	100%	100%	100%	100%	100%
11365	15	V131V	NSP6	0%	0%	5%	0%	100%	0%	0%	0%	0%	0%	0%	0%
11572	6	F200F	NSP6	0%	0%	0%	0%	0%	0%	0%	0%	86%	0%	0%	0%
12070	6	L76L	NSP7	0%	3%	0%	0%	0%	0%	0%	0%	0%	0%	100%	0%
12718	14	Q11H	NSP9	0%	0%	0%	0%	100%	0%	0%	0%	0%	0%	0%	0%
12880	9	I65I	NSP9	0%	0%	0%	50%	0%	0%	0%	0%	0%	0%	0%	0%
13168	9	H48H	NSP10	0%	0%	0%	0%	0%	0%	100%	0%	0%	0%	0%	0%
13339	6	N105N	NSP10	0%	0%	0%	0%	0%	0%	0%	0%	0%	100%	0%	0%
14408	105	P314L	NSP12b	53%	48%	52%	61%	57%	77%	78%	29%	86%	67%	80%	50%
14925	6	V486V	NSP12b	0%	0%	0%	0%	0%	0%	0%	86%	0%	0%	0%	0%
15451	184	G662S	NSP12b	100%	100%	100%	100%	100%	100%	100%	100%	100%	100%	100%	100%
15882	14	S805S	NSP12b	0%	0%	0%	0%	100%	0%	0%	0%	0%	0%	0%	0%
15952	46	L829I	NSP12b	100%	0%	0%	0%	0%	0%	0%	14%	0%	0%	0%	0%
16389	6	N51N	NSP13	0%	0%	0%	0%	0%	0%	67%	0%	0%	0%	0%	0%
16466	182	P77L	NSP13	100%	97%	95%	100%	100%	100%	100%	100%	100%	83%	100%	100%
16726	10	H164Y	NSP13	0%	3%	0%	6%	0%	0%	89%	0%	0%	0%	0%	0%
16887	10	Y217Y	NSP13	0%	0%	5%	50%	0%	0%	0%	0%	0%	0%	0%	0%
17040	5	N268N	NSP13	0%	0%	5%	0%	0%	0%	0%	0%	0%	0%	0%	100%
17236	7	I334V	NSP13	0%	0%	0%	0%	0%	0%	0%	0%	100%	0%	0%	0%
17271	6	K345K	NSP13	0%	0%	0%	0%	0%	0%	0%	0%	86%	0%	0%	0%
17634	6	D466E	NSP13	0%	0%	0%	0%	0%	0%	0%	0%	86%	0%	0%	0%
17676	12	I480I	NSP13	0%	0%	0%	0%	0%	92%	0%	0%	0%	0%	0%	0%
17937	9	R567R	NSP13	0%	0%	0%	50%	0%	0%	0%	0%	0%	0%	0%	0%
18294	9	A85A	NSP14	0%	0%	0%	50%	0%	0%	0%	0%	0%	0%	0%	0%
18492	11	P151P	NSP14	0%	0%	0%	0%	0%	85%	0%	0%	0%	0%	0%	0%
18630	4	Y197Y	NSP14	0%	0%	0%	0%	0%	0%	0%	0%	0%	0%	0%	100%
18647	6	P203L	NSP14	0%	0%	0%	0%	0%	0%	67%	0%	0%	0%	0%	0%
18744	31	Y235Y	NSP14	67%	0%	5%	0%	0%	0%	0%	0%	0%	0%	0%	0%
19220	182	A394V	NSP14	100%	100%	100%	100%	100%	100%	100%	100%	100%	100%	100%	100%
19524	12	L495L	NSP14	0%	0%	5%	0%	0%	0%	0%	0%	0%	100%	100%	0%

19859	8	A80V	NSP15		0%	0%	0%	0%	0%	0%	89%	0%	0%	0%	0%	0%
20468	6	A283V	NSP15		0%	0%	0%	0%	0%	0%	67%	0%	0%	0%	0%	0%
20995	5	G113C	NSP16		0%	0%	0%	0%	0%	0%	0%	0%	0%	0%	100%	0%
21137	7	K160R	NSP16		0%	0%	0%	0%	0%	0%	0%	100%	0%	0%	0%	0%
21169	6	I171V	NSP16		0%	0%	0%	0%	0%	0%	0%	0%	86%	0%	0%	0%
21381	9	S241S	NSP16		0%	0%	0%	50%	0%	0%	0%	0%	0%	0%	0%	0%
21595	5	V11V	S		0%	0%	0%	0%	0%	0%	0%	0%	0%	0%	100%	0%
21618	185	T19R	S		100%	100%	100%	100%	100%	100%	100%	100%	100%	100%	100%	100%
21668	4	V36F	S		0%	0%	0%	0%	0%	0%	0%	0%	0%	0%	0%	100%
21811	10	V83V	S		2%	0%	0%	50%	0%	0%	0%	0%	0%	0%	0%	0%
21846	73	T95I	S		0%	0%	100%	94%	0%	100%	100%	100%	0%	0%	0%	100%
21987	184	G142D	S		100%	100%	100%	94%	100%	100%	100%	100%	100%	100%	100%	100%
21995	4	Y145H	S		0%	0%	0%	0%	0%	0%	0%	0%	0%	0%	0%	100%
22227	7	A222V	S		0%	0%	0%	0%	0%	0%	0%	0%	0%	0%	0%	100%
22314	5	P251L	S		0%	0%	0%	0%	0%	0%	0%	0%	0%	0%	100%	0%
22564	5	N334N	S		0%	0%	0%	0%	0%	0%	0%	0%	0%	0%	100%	0%
22880	3	N440Y	S		0%	0%	0%	0%	0%	0%	0%	0%	0%	50%	0%	0%
22917	185	L452R	S		100%	100%	100%	100%	100%	100%	100%	100%	100%	100%	100%	100%
22995	185	T478K	S		100%	100%	100%	100%	100%	100%	100%	100%	100%	100%	100%	100%
23403	185	D614G	S		100%	100%	100%	100%	100%	100%	100%	100%	100%	100%	100%	100%
23557	7	P665P	S		0%	0%	0%	0%	0%	0%	78%	0%	0%	0%	0%	0%
23604	99	P681R	S		51%	45%	33%	61%	57%	62%	78%	43%	86%	50%	100%	50%
23625	8	A688V	S		0%	3%	0%	0%	50%	0%	0%	0%	0%	0%	0%	0%
24110	5	I850L	S		0%	0%	0%	0%	0%	0%	0%	71%	0%	0%	0%	0%
24208	7	I882I	S		0%	0%	0%	0%	0%	0%	0%	0%	100%	0%	0%	0%
24410	185	D950N	S		100%	100%	100%	100%	100%	100%	100%	100%	100%	100%	100%	100%
24904	6	I1114I	S		0%	0%	0%	0%	0%	0%	0%	0%	0%	100%	0%	0%
24921	5	T1120I	S		0%	0%	0%	0%	0%	0%	0%	0%	0%	83%	0%	0%
25439	6	K16T	ORF3a		0%	0%	0%	0%	0%	0%	0%	86%	0%	0%	0%	0%
25445	14	G18V	ORF3a		0%	0%	0%	0%	100%	0%	0%	0%	0%	0%	0%	0%
25459	13	A23S	ORF3a		0%	0%	0%	0%	93%	0%	0%	0%	0%	0%	0%	0%
25469	179	S26L	ORF3a		98%	100%	86%	89%	100%	100%	100%	100%	100%	100%	100%	100%
25496	6	I35T	ORF3a		0%	0%	0%	0%	0%	0%	0%	0%	86%	0%	0%	0%
25538	13	G49V	ORF3a		0%	0%	0%	0%	0%	100%	0%	0%	0%	0%	0%	0%
25563	10	Q57H	ORF3a		2%	0%	0%	50%	0%	0%	0%	0%	0%	0%	0%	0%
25614	4	S74S	ORF3a		0%	0%	0%	0%	0%	0%	0%	0%	0%	0%	0%	100%
25626	12	H78H	ORF3a		0%	0%	0%	0%	0%	92%	0%	0%	0%	0%	0%	0%
25654	3	V88L	ORF3a		4%	0%	0%	0%	0%	0%	0%	0%	0%	0%	0%	0%
25714	7	L108F	ORF3a		0%	0%	0%	0%	0%	0%	0%	14%	0%	100%	0%	0%
25996	9	V202L	ORF3a		0%	0%	0%	0%	0%	0%	100%	0%	0%	0%	0%	0%
26054	13	T221K	ORF3a		0%	0%	0%	0%	0%	100%	0%	0%	0%	0%	0%	0%
26256	9	F4F	E		0%	0%	0%	50%	0%	0%	0%	0%	0%	0%	0%	0%
26681	9	F53F	M		0%	0%	0%	0%	0%	0%	0%	0%	0%	33%	100%	0%
26767	185	I82T	M		100%	100%	100%	100%	100%	100%	100%	100%	100%	100%	100%	100%
26906	30	I128I	M		13%	21%	10%	22%	0%	31%	11%	14%	0%	67%	0%	25%
27291	7	D30D	ORF6		0%	0%	0%	0%	0%	0%	0%	0%	100%	0%	0%	0%
27527	29	P45L	ORF7a		0%	86%	0%	0%	0%	0%	0%	0%	0%	0%	0%	100%
27638	163	V82A	ORF7a		100%	90%	100%	100%	0%	100%	100%	43%	100%	100%	100%	75%
27752	185	T120I	ORF7a		100%	100%	100%	100%	100%	100%	100%	100%	100%	100%	100%	100%
27762	3	E3*	ORF7b		0%	0%	0%	0%	0%	0%	0%	0%	0%	0%	0%	75%
27874	181	T40I	ORF7b		100%	100%	100%	100%	100%	100%	100%	86%	100%	100%	100%	100%
27916	12	G8E	ORF8		0%	0%	0%	0%	0%	92%	0%	0%	0%	0%	0%	0%
28086	5	A65S	ORF8		0%	0%	0%	0%	0%	0%	0%	0%	0%	0%	100%	0%
28299	45	Q9L	N		100%	0%	0%	0%	0%	0%	0%	0%	0%	0%	0%	0%
28378	31	A35A	N		33%	10%	0%	0%	0%	100%	0%	0%	0%	0%	0%	0%
28396	8	R41R	N		0%	0%	0%	0%	0%	0%	89%	0%	0%	0%	0%	0%
28461	183	D63G	N		100%	100%	95%	100%	100%	100%	100%	100%	100%	100%	100%	75%
28724	7	P151S	N		0%	0%	0%	0%	0%	54%	0%	0%	0%	0%	0%	0%
28816	14	Q181Q	N		0%	0%	0%	0%	100%	0%	0%	0%	0%	0%	0%	0%
28846	5	R191R	N		0%	0%	0%	0%	0%	0%	56%	0%	0%	0%	0%	0%
28881	185	R203M	N		100%	100%	100%	100%	100%	100%	100%	100%	100%	100%	100%	100%
28916	179	G215C	N		100%	100%	100%	100%	100%	100%	78%	100%	100%	83%	100%	100%
29062	6	T263T	N		0%	0%	0%	0%	0%	0%	67%	0%	0%	0%	0%	0%
29081	5	V270L	N		0%	0%	0%	0%	0%	0%	0%	0%	0%	83%	0%	0%
29402	185	D377Y	N		100%	100%	100%	100%	100%	100%	100%	100%	100%	100%	100%	100%
29537	3	29537	3'UTR		0%	0%	0%	0%	0%	0%	0%	0%	0%	0%	0%	75%
29614	8	C19C	ORF10		0%	0%	0%	0%	0%	0%	0%	0%	86%	0%	0%	0%
29742	185	29742	3'UTR		100%	100%	100%	100%	100%	100%	100%	100%	100%	100%	100%	100%
