# Supplementary material for: Introduction, Spread and Impact of the SARS-CoV-2 Omicron Variants BA.1 and BA.2 in Cyprus
Source: Microorganisms. 2022 Aug 23;10(9):1688. doi: 10.3390/microorganisms10091688 (PMC9503937; doi:10.3390/microorganisms10091688)
Supplement: Supplementary file 1 [file microorganisms-10-01688-s001.zip › Supplementary Table S2.pdf]

Table S2. Mutations identified in the Omicron sub-lineages in Cyprus between November 2021 and April 2022. Only SNPs with frequency >50% were included. Mutations with frequency >75% are marked in green, SNPs with frequency between 50% and 75% are marked in yellow.

Position	Variant	protein		BA.1.1	BA.1	BA.1.17.2	BA.1.17	BA.1.15	BA.1.1.1	BA.1.1.15	BA.1.15.1	BA.1.16	BA.1.10	BA.1.18	BA.1.19	BA.1.20	BA.1.17.1	BA.2	BA.2.9	BA.2.37	BA.2.3	BA.2.1	BA.2.25.1
84	84	5'UTR		0%	0%	0%	0%	0%	0%	0%	0%	0%	0%	0%	0%	0%	0%	0%	0%	0%	100%	0%	0%
188	188	5'UTR		0%	0%	0%	0%	0%	0%	0%	0%	0%	0%	50%	0%	0%	0%	1%	0%	0%	0%	0%	0%
241	241	5'UTR		100%	100%	100%	100%	100%	100%	100%	100%	100%	100%	100%	100%	100%	100%	97%	93%	100%	100%	100%	100%
670	S135R	NSP1		0%	0%	0%	0%	0%	0%	0%	0%	0%	0%	0%	0%	0%	0%	100%	100%	100%	100%	100%	100%
1585	G260G	NSP2		0%	0%	0%	0%	0%	0%	0%	0%	0%	0%	0%	0%	0%	0%	0%	67%	0%	0%	0%	0%
2470	A555A	NSP2		60%	0%	0%	0%	0%	0%	100%	0%	0%	0%	0%	0%	0%	0%	1%	0%	0%	0%	0%	0%
2790	T24I	NSP3		0%	0%	0%	0%	0%	0%	0%	0%	0%	0%	0%	0%	0%	0%	100%	100%	100%	100%	100%	100%
2832	K38R	NSP3		99%	100%	100%	100%	100%	100%	100%	100%	100%	100%	100%	100%	100%	100%	0%	0%	0%	0%	0%	0%
3037	F106F	NSP3		100%	100%	98%	100%	100%	100%	100%	100%	100%	100%	100%	100%	100%	100%	100%	100%	100%	100%	100%	100%
3104	Y129H	NSP3		0%	0%	0%	0%	0%	0%	0%	0%	100%	0%	0%	0%	0%	0%	0%	0%	0%	0%	0%	0%
3241	D174D	NSP3		0%	0%	98%	0%	0%	0%	0%	0%	0%	0%	0%	0%	0%	0%	0%	0%	0%	0%	0%	0%
4184	G489S	NSP3		0%	0%	0%	0%	0%	0%	0%	0%	0%	0%	0%	0%	0%	0%	100%	100%	100%	100%	100%	100%
4321	A534A	NSP3		0%	0%	0%	0%	6%	0%	0%	0%	0%	0%	0%	0%	0%	0%	100%	100%	100%	100%	100%	100%
5386	A889A	NSP3		99%	93%	100%	100%	88%	100%	100%	83%	100%	100%	100%	50%	50%	100%	0%	0%	0%	0%	0%	0%
5730	T1004I	NSP3		0%	0%	0%	0%	0%	0%	0%	0%	0%	0%	100%	0%	0%	0%	0%	0%	0%	0%	0%	0%
5924	V1069I	NSP3		1%	0%	94%	100%	0%	0%	0%	0%	0%	0%	0%	0%	0%	100%	0%	0%	0%	0%	0%	0%
6070	I1117I	NSP3		0%	7%	0%	0%	0%	0%	0%	0%	0%	50%	0%	50%	0%	0%	0%	0%	0%	0%	0%	0%
7300	A1527A	NSP3		0%	0%	0%	0%	0%	0%	0%	0%	0%	0%	0%	0%	0%	0%	0%	0%	0%	100%	0%	0%
8393	A1892T	NSP3		100%	88%	96%	100%	75%	100%	100%	83%	100%	0%	100%	50%	50%	100%	0%	0%	0%	0%	0%	0%
8991	A146V	NSP4		0%	0%	0%	0%	0%	0%	0%	0%	0%	0%	0%	0%	0%	0%	0%	0%	0%	100%	0%	0%
9344	L264F	NSP4		1%	0%	0%	0%	0%	0%	0%	0%	0%	0%	0%	0%	0%	0%	100%	100%	100%	100%	100%	100%
9424	V290V	NSP4		0%	0%	0%	0%	0%	0%	0%	0%	0%	0%	0%	0%	0%	0%	99%	93%	100%	100%	100%	100%
9534	T327I	NSP4		0%	0%	0%	0%	0%	0%	0%	0%	0%	0%	0%	0%	0%	0%	99%	100%	100%	100%	100%	100%
9866	L438F	NSP4		0%	0%	0%	0%	0%	0%	0%	0%	0%	0%	0%	0%	0%	0%	100%	100%	100%	100%	100%	100%
10029	T492I	NSP4		100%	98%	98%	100%	100%	100%	100%	100%	100%	100%	100%	100%	100%	100%	100%	100%	100%	100%	100%	100%
10135	L27L	NSP5		0%	0%	0%	0%	100%	0%	0%	100%	0%	0%	0%	0%	0%	0%	0%	0%	0%	0%	0%	0%
10138	N28N	NSP5		0%	7%	0%	0%	0%	0%	9%	0%	0%	0%	0%	0%	0%	0%	0%	0%	100%	0%	0%	0%
10198	D48D	NSP5		1%	0%	0%	0%	0%	0%	0%	0%	0%	0%	0%	0%	0%	0%	100%	100%	100%	100%	100%	100%
10447	R131R	NSP5		0%	0%	0%	0%	0%	0%	0%	0%	0%	0%	0%	0%	0%	0%	100%	100%	100%	100%	100%	100%
10449	P132H	NSP5		100%	92%	98%	100%	75%	100%	100%	83%	100%	0%	100%	100%	50%	100%	99%	100%	100%	100%	100%	100%
11537	I189V	NSP6		99%	100%	100%	100%	100%	100%	100%	100%	100%	100%	100%	100%	100%	100%	0%	0%	0%	0%	0%	0%
12747	T21I	NSP9		0%	0%	0%	0%	0%	0%	0%	0%	0%	0%	0%	0%	0%	0%	0%	0%	0%	100%	0%	0%
12880	I65I	NSP9		0%	0%	0%	0%	0%	0%	0%	0%	0%	0%	0%	0%	0%	0%	100%	100%	100%	100%	100%	100%
13195	V57V	NSP10		100%	97%	100%	100%	100%	100%	100%	100%	100%	100%	100%	50%	100%	100%	0%	0%	0%	0%	0%	0%
13570	G35S	NSP12b		0%	0%	0%	0%	0%	0%	100%	0%	0%	0%	0%	0%	0%	0%	0%	0%	0%	0%	0%	0%
14014	F183L	NSP12b		0%	2%	0%	0%	88%	0%	0%	17%	0%	0%	0%	0%	0%	0%	0%	0%	0%	0%	0%	0%
14117	T217M	NSP12b		0%	0%	0%	0%	0%	0%	100%	0%	0%	0%	0%	0%	0%	0%	0%	0%	0%	0%	0%	0%
14408	P314L	NSP12b		73%	75%	86%	90%	88%	92%	73%	100%	67%	100%	100%	100%	100%	0%	84%	80%	100%	100%	0%	0%
15240	N591N	NSP12b		100%	100%	98%	100%	100%	100%	100%	100%	100%	100%	100%	100%	100%	100%	0%	13%	0%	0%	0%	0%
15714	L749L	NSP12b		0%	0%	0%	0%	0%	0%	0%	0%	0%	0%	0%	0%	0%	0%	100%	100%	100%	100%	100%	100%
16064	Q866R	NSP12b		0%	0%	0%	0%	0%	100%	0%	0%	0%	0%	0%	0%	0%	0%	0%	0%	0%	0%	0%	0%
17410	R392C	NSP13		0%	0%	0%	0%	0%	0%	0%	0%	0%	0%	0%	0%	0%	0%	100%	100%	100%	100%	100%	100%
18163	I42V	NSP14		99%	88%	94%	100%	75%	100%	100%	83%	100%	0%	100%	0%	50%	100%	100%	100%	100%	100%	100%	100%
19524	L495L	NSP14		0%	0%	0%	0%	0%	0%	0%	0%	0%	0%	0%	0%	0%	0%	0%	0%	0%	100%	0%	0%
19955	T112I	NSP15		0%	0%	0%	0%	0%	0%	0%	0%	0%	0%	0%	0%	0%	0%	95%	87%	100%	100%	100%	100%
20055	E145E	NSP15		0%	0%	0%	0%	0%	0%	0%	0%	0%	0%	0%	0%	0%	0%	100%	100%	100%	100%	100%	100%
21618	T19I	S		0%	0%	0%	0%	0%	0%	0%	0%	0%	0%	0%	0%	0%	0%	98%	100%	100%	100%	100%	100%
21762	A67V	S		100%	100%	100%	100%	100%	100%	100%	100%	100%	100%	100%	100%	100%	100%	7%	0%	0%	0%	100%	100%
21846	T95I	S		99%	88%	94%	100%	75%	100%	100%	83%	100%	0%	100%	0%	50%	100%	7%	0%	0%	0%	100%	100%
21987	G142D	S		0%	0%	0%	0%	0%	0%	0%	0%	0%	0%	0%	0%	0%	0%	100%	100%	100%	100%	100%	100%
22200	V213G	S		0%	0%	0%	0%	0%	0%	0%	0%	0%	0%	0%	0%	0%	0%	100%	100%	100%	100%	100%	100%
22295	H245N	S		0%	0%	0%	0%	0%	0%	0%	0%	0%	0%	0%	0%	0%	0%	0%	0%	0%	100%	0%	0%

22578	G339D	S		99%	88%	94%	100%	75%	100%	100%	83%	100%	0%	100%	0%	50%	100%	100%	100%	100%	100%	100%	100%
22599	R346K	S		98%	0%	0%	0%	0%	100%	100%	0%	0%	0%	0%	0%	0%	0%	0%	0%	0%	0%	0%	0%
22673	S371L	S		99%	100%	100%	100%	100%	100%	100%	100%	100%	100%	100%	100%	100%	100%	0%	0%	0%	0%	0%	0%
22674	S371F	S		1%	0%	0%	0%	0%	0%	0%	0%	0%	0%	0%	0%	0%	0%	100%	100%	100%	100%	100%	100%
22679	S373P	S		100%	100%	100%	100%	100%	100%	100%	100%	100%	100%	100%	100%	100%	100%	100%	100%	100%	100%	100%	100%
22686	S375F	S		100%	100%	100%	100%	100%	100%	100%	100%	100%	100%	100%	100%	100%	100%	100%	100%	100%	100%	100%	100%
22688	T376A	S		1%	0%	0%	0%	0%	0%	0%	0%	0%	0%	0%	0%	0%	0%	100%	100%	100%	100%	100%	100%
22775	D405N	S		1%	0%	0%	0%	0%	0%	0%	0%	0%	0%	0%	0%	0%	0%	100%	100%	100%	100%	100%	100%
22786	R408S	S		1%	0%	0%	0%	0%	0%	0%	0%	0%	0%	0%	0%	0%	0%	100%	100%	100%	100%	100%	100%
22813	K417N	S		100%	100%	100%	100%	100%	100%	100%	100%	100%	100%	100%	100%	100%	100%	100%	100%	100%	100%	100%	100%
22882	N440K	S		100%	100%	100%	100%	100%	100%	100%	100%	100%	100%	100%	100%	100%	100%	100%	100%	100%	100%	100%	100%
22898	G446S	S		100%	100%	100%	100%	100%	100%	100%	100%	100%	100%	100%	100%	100%	100%	0%	0%	0%	0%	0%	0%
22992	S477N	S		100%	93%	100%	100%	100%	100%	100%	100%	100%	100%	100%	100%	100%	100%	100%	100%	100%	100%	100%	100%
22995	T478K	S		100%	93%	100%	100%	100%	100%	100%	100%	100%	100%	100%	100%	100%	100%	100%	100%	100%	100%	100%	100%
23013	E484A	S		100%	93%	100%	100%	100%	100%	100%	100%	100%	100%	100%	100%	100%	100%	100%	100%	100%	100%	100%	100%
23040	Q493R	S		100%	93%	100%	100%	100%	100%	100%	100%	100%	100%	100%	100%	100%	100%	100%	100%	100%	100%	100%	100%
23048	G496S	S		100%	93%	100%	100%	100%	100%	100%	100%	100%	100%	100%	100%	100%	100%	0%	0%	0%	0%	0%	0%
23055	Q498R	S		100%	93%	100%	100%	100%	100%	100%	100%	100%	100%	100%	100%	100%	100%	100%	100%	100%	100%	100%	100%
23063	N501Y	S		100%	93%	100%	100%	100%	100%	100%	100%	100%	100%	100%	100%	100%	100%	100%	100%	100%	100%	100%	100%
23075	Y505H	S		100%	93%	100%	100%	100%	100%	100%	100%	100%	100%	100%	100%	100%	100%	100%	100%	100%	100%	100%	100%
23202	T547K	S		99%	92%	98%	100%	94%	100%	100%	83%	100%	50%	100%	0%	50%	100%	0%	0%	0%	0%	0%	0%
23403	D614G	S		99%	98%	100%	100%	100%	100%	100%	100%	100%	100%	100%	100%	100%	100%	100%	100%	100%	100%	100%	100%
23525	H655Y	S		88%	92%	90%	100%	94%	83%	100%	100%	67%	100%	100%	50%	100%	100%	96%	93%	100%	100%	100%	100%
23599	N679K	S		83%	88%	90%	95%	81%	83%	100%	100%	67%	50%	100%	0%	100%	100%	94%	93%	100%	100%	100%	100%
23604	P681H	S		83%	86%	90%	95%	81%	83%	100%	100%	67%	50%	100%	0%	100%	100%	93%	93%	100%	100%	100%	100%
23664	A701V	S		0%	0%	96%	0%	0%	0%	0%	0%	0%	0%	0%	0%	0%	0%	0%	0%	0%	0%	0%	0%
23854	N764K	S		100%	100%	100%	100%	100%	100%	100%	100%	100%	100%	100%	100%	100%	100%	100%	100%	100%	100%	100%	100%
23948	D796Y	S		100%	100%	100%	100%	100%	100%	100%	100%	100%	100%	100%	100%	100%	100%	100%	100%	100%	100%	100%	100%
24130	N856K	S		93%	100%	96%	100%	100%	100%	100%	100%	100%	100%	100%	100%	100%	100%	1%	0%	0%	0%	0%	0%
24424	Q954H	S		100%	100%	100%	100%	100%	100%	100%	100%	100%	100%	100%	100%	100%	100%	100%	100%	100%	100%	100%	100%
24469	N969K	S		100%	100%	100%	100%	100%	100%	100%	100%	100%	100%	100%	100%	100%	100%	100%	100%	100%	100%	100%	100%
24503	L981F	S		100%	100%	100%	100%	100%	100%	100%	100%	100%	100%	100%	100%	100%	100%	0%	0%	0%	0%	0%	0%
24803	I1081V	S		0%	0%	0%	0%	0%	0%	0%	83%	0%	0%	0%	0%	0%	0%	0%	0%	0%	0%	0%	0%
25000	D1146D	S		100%	100%	100%	100%	100%	100%	100%	100%	100%	100%	100%	100%	100%	100%	100%	100%	100%	100%	100%	100%
25416	F8F	ORF3a		0%	0%	0%	0%	0%	0%	0%	0%	0%	0%	0%	0%	0%	0%	38%	0%	100%	0%	0%	0%
25584	T64T	ORF3a		100%	98%	100%	100%	100%	100%	100%	100%	100%	100%	100%	100%	100%	100%	100%	100%	100%	100%	100%	100%
25624	H78Y	ORF3a		0%	0%	0%	0%	0%	0%	0%	0%	0%	0%	0%	0%	0%	0%	0%	100%	0%	0%	0%	0%
25708	L106F	ORF3a		0%	0%	0%	0%	100%	0%	0%	100%	0%	0%	0%	0%	0%	0%	0%	0%	0%	0%	0%	0%
25810	L140F	ORF3a		0%	0%	0%	0%	0%	0%	0%	0%	0%	0%	0%	0%	0%	0%	0%	0%	0%	100%	0%	0%
25855	D155Y	ORF3a		0%	0%	0%	0%	0%	0%	0%	0%	0%	0%	100%	0%	0%	0%	0%	0%	0%	0%	0%	0%
26060	T223I	ORF3a		0%	0%	2%	0%	0%	0%	9%	0%	0%	0%	0%	0%	0%	0%	100%	100%	100%	100%	100%	100%
26270	T9I	E		100%	98%	98%	100%	94%	100%	100%	100%	100%	100%	100%	100%	100%	100%	100%	100%	100%	100%	100%	100%
26530	D3G	M		100%	100%	100%	100%	100%	100%	100%	100%	100%	100%	100%	100%	100%	100%	0%	0%	0%	0%	0%	0%
26577	Q19E	M		100%	97%	100%	100%	88%	100%	100%	100%	100%	0%	100%	100%	100%	100%	100%	100%	100%	100%	100%	100%
26709	A63T	M		99%	90%	94%	100%	75%	100%	100%	83%	100%	50%	100%	0%	50%	100%	100%	100%	100%	100%	100%	100%
26858	F112F	M		0%	0%	0%	0%	0%	0%	0%	0%	0%	0%	0%	0%	0%	0%	100%	100%	100%	100%	100%	100%
27259	M19M	ORF6		100%	98%	100%	100%	100%	100%	100%	100%	100%	100%	100%	100%	100%	100%	100%	100%	100%	100%	100%	100%
27382	D61L	ORF6		0%	0%	0%	0%	0%	8%	0%	0%	0%	0%	0%	0%	0%	0%	100%	100%	100%	100%	100%	100%
27807	L17L	ORF7b		95%	98%	98%	100%	100%	100%	100%	100%	100%	100%	100%	100%	100%	100%	100%	100%	100%	100%	100%	100%
28271	28271	3'UTR		100%	100%	100%	100%	100%	100%	100%	100%	100%	100%	100%	100%	100%	100%	100%	100%	100%	100%	100%	100%
28311	P13L	N		98%	98%	100%	100%	100%	100%	100%	100%	100%	100%	100%	100%	100%	100%	100%	100%	100%	100%	100%	100%
28881	RG203KR	N		91%	98%	98%	100%	100%	100%	91%	100%	100%	100%	100%	100%	100%	100%	98%	100%	100%	100%	100%	100%
29301	D343G	N		0%	2%	0%	0%	75%	0%	0%	83%	0%	0%	0%	0%	0%	0%	0%	0%	0%	0%	0%	0%
29510	S413R	N		0%	0%	0%	0%	0%	0%	0%	0%	0%	0%	0%	0%	0%	0%	88%	100%	100%	100%	100%	100%
29632	N25N	ORF10		0%	59%	0%	5%	0%	0%	0%	0%	100%	0%	0%	0%	0%	0%	0%	0%	0%	0%	0%	0%
29755	29755	3'UTR		0%	0%	0%	0%	0%	0%	100%	0%	0%	0%	0%	0%	0%	0%	1%	0%	0%	0%	0%	0%
